# Parenting Style and Child Internet Addiction in China: Mediation Effect of Parental Active Meditation

**DOI:** 10.3390/ijerph22040461

**Published:** 2025-03-21

**Authors:** Shu-Wen Liu, Sheng Chen, Chienchung Huang, Julia Vallario

**Affiliations:** 1 Faculty of Health Sciences, University of Saint Joseph, Macau, China; helen.liu@usj.edu.mo; 2 School of Public Administration, Guangdong University of Foreign Studies, Guangzhou 510420, China; 3 School of Social Work, Rutgers University, New Brunswick, NJ 08901, USA; jv801@scarletmail.rutgers.edu

**Keywords:** active mediation, authoritative parenting, authoritarian parenting, internet addiction, permissive parenting

## Abstract

With the rapid advancement of technology, internet addiction among children has become a growing concern, particularly in China. This study examines the impact of different parenting styles on internet addiction and the role of parental mediation on the relationship among Chinese children. A survey of 600 Chinese families with children aged 6 to 17 was conducted in 2024. Using structural equation modeling (SEM), the results reveal that authoritative parenting is linked to lower levels of internet addiction, while authoritarian and permissive parenting are associated with higher levels. Parental mediation partly mediates this relationship, with authoritative parents who are actively involved with their children’s internet activities showing a reduced risk of addiction. The findings highlight that both parenting style and parental intervention are key factors in addressing internet addiction among Chinese children. Encouraging authoritative parenting alongside active mediation may help children mitigate internet addiction.

## 1. Introduction

The rapid advancement of the internet and smartphones has revolutionized daily life, making them indispensable tools for communication, information gathering, academic research, and entertainment [1,2,3]. Connecting with peers online is increasingly becoming a part of the lives of children and adolescents, as they use the internet and smart phones for both academic and social purposes [3]. Indeed, technology has become a part of children and adolescents in daily activities, from social media usage to educational purposes [1,4].

While these technologies offer numerous advantages, concerns have arisen regarding their excessive use, particularly among children and adolescents [5,6,7]. Excessive internet use, commonly referred to as internet addiction, has been linked to a variety of psychological and behavioral issues, including mental health symptoms, academic struggles, and deteriorating social relationships [8,9,10]. This growing concern has become especially pronounced in China, where internet addiction has been documented as adverse effects on children’s academic performance, social well-being, and mental health, highlighting issues such as anxiety, strained familial and peer relationships, and even substance abuse among children and adolescents [11,12].

Parenting style, a concept referring to the general patterns of parental attitudes and behaviors toward their children, is a critical factor in children’s emotional and psychological development [13,14]. Commonly categorized into three types—authoritarian, authoritative, and permissive—parenting styles have been shown to influence a range of outcomes, including life satisfaction, academic adjustment, and, importantly, susceptibility to internet addiction [15,16]. Authoritative parenting, which combines nurture and structure, has been associated with constructive outcomes such as advanced academic achievement and healthier emotional regulation, thus reducing the likelihood of developing unhealthy internet habits [17,18,19]. Conversely, authoritarian parenting, which focuses on discipline and control, and permissive parenting, which emphasizes emotional connection and responsiveness over strict rules and discipline on connections, have shown to increase internet addiction, as they either overly restrict or fail to monitor children’s internet activities [20,21]. This nuanced understanding underscores the importance of parenting styles in fostering healthy developmental trajectories in the digital age.

Cultural differences may play a significant role in how parenting styles manifest, and in China, parental mediation and family dynamics differ from those in Western countries [22,23]. Chinese parenting styles, influenced by Confucian traditions, emphasize conformity, psychological control, and family obligation more than Western parenting, which typically promotes autonomy and emotional expression [22,24]. In addition, in contrast to Western parenting, which values open communication, Chinese parenting tends to rely on covert monitoring and hierarchical family structures, reinforcing a strong sense of filial piety and interdependence [23,25]. However, despite the growing concern about internet addiction among Chinese children, limited research has explored the specific mechanisms that link parenting style to this phenomenon in the Chinese context, especially for young children. This study aims to investigate the relationship between parenting style and child internet addiction in China, with a particular focus on the mediating role of parental mediation. There are three objectives: 1. To examine the influence of different parenting styles on child internet addiction. 2. To explore the role of parental mediation in shaping children’s internet use behaviors. 3. To analyze whether parental mediation serves as a mediator between parenting style and child internet addiction. The research questions of this study are as follows: 1. How do different parenting styles influence child internet addiction in China? 2. What role does parental mediation play in children’s internet use behaviors? 3. Does parental mediation mediate the relationship between parenting style and child internet addiction?

## 2. Literature Review

### 2.1. Parenting Style and Internet Addiction

Research on internet addiction among children and adolescents has increasingly concentrated on the roles of parenting style [10,13,15]. Authoritative parenting typically acts as a preservative factor against internet addiction. Studies suggest that the supportive domain and structured self-governance provided by authoritative parents empower children and adolescents to prosper in self-discipline and responsibility, alleviating the risk of internet addiction [14,18]. Inversely, authoritarian and permissive styles, marked by a lack of nurturing and disorderly conduct are associated with higher risks of internet addictions [21,24]. Research indicates that authoritarian parenting may lead to higher instances of defiant or avoidant behaviors online, while permissive parenting can encourage excessive screen time due to the lack of boundaries [5,6]. Specifically, Liu and Li [21] used 2758 seventh to ninth graders and found that authoritative parenting was negatively associated with internet addiction (r = −0.21), while authoritarian and permissive parenting were both positively associated with internet addiction (r = 0.29 and 0.27, respectively). Zhang et al. [26] used 660 Chinese middle-school adolescents and found that authoritative parenting was negatively associated with internet addiction (r = −0.19) while authoritarian and permissive parenting were both positively associated with internet addiction (r = 0.25 and 0.29, respectively).

Similarly, Zhang et al. [19] used 947 students from grades 7 to 9 to investigate the relationship between different parenting styles and internet addiction in China. The study found that students with parents who displayed authoritative styles were associated with having lower levels of internet addiction. Guo et al. [20] found that positive parenting, such as emotional warmth, was a protective factor against internet addiction, while negative parenting, characterized by rejection and overprotection, increased the risk of addiction. Li et al. [27] also found that parental positive support reduced the risk of internet addiction, while negative control, such as excessive restriction or authoritarian behaviors, increased it. In short, the findings from empirical studies align with the broader understanding that authoritative parenting, which balances warmth and discipline, promotes healthier behaviors in adolescents and reduces internet addiction, while authoritarian and permissive parenting, represented by parental refusal or neglect, are related to increased internet addiction.

### 2.2. Parental Mediation and Internet Addiction

Parental mediation refers to the strategies parents use to monitor, guide, and control their children’s internet use. Parents can avoid the negative effects of child internet addiction by proactively intervening in child online behaviors. Parental mediation plays a critical role in diminishing internet addiction. Active mediation, where parents discuss online content and encourage critical thinking, has shown a strong association with reduced internet addiction, specifically when utilized with an authoritative parenting approach [28,29,30]. Li et al. [17] used 3026 school children aged 9–14 years and explored the role of parental mediation and parent–child relationships in adolescents’ internet addiction. The study found that active parental mediation, where parents engage in discussions with their children about internet use, was associated with lower levels of internet addiction (beta = −0.21). Ren and Zhu [31] used 1284 middle school students in China and found that the effectiveness of parental mediation strategies varies depending on the overall parenting style. Supportive parents who engage in active mediation tend to have children with healthier internet use habits.

Succinctly, the literature reviewed demonstrates that parenting styles and parental mediation play significant roles in the risk of internet addiction. Authoritative parenting is associated with lower levels of internet addiction, while authoritarian and permissive parenting increase the risk. Parental active mediation is effective in reducing internet addiction, especially when combined with supportive parent–child relationships. Future research should continue to explore the interplay between parenting styles and parental mediation in the development of internet addiction.

## 3. Conceptual Framework and Hypotheses

Bronfenbrenner’s bioecological theory of human development [32,33] provides the conceptual framework for this study to examine the influence of parenting styles and parental mediation on child internet addiction. According to Bronfenbrenner, child development occurs within a set of nested systems, with family being a key microsystem that directly shapes the child’s behavior. Parenting styles, as core family processes, play a crucial role in setting the tone for child behaviors, including their internet activities. Additionally, parental mediation acts as a mediating factor in this relationship. By integrating these elements, the framework explores how authoritative, authoritarian, and permissive parenting affect mediation strategies, which then impact the risk of internet addiction in children. This approach, rooted in the bioecological theory, underscores the complex interplay between family processes in shaping child digital behavior. The following hypotheses are proposed based on Bronfenbrenner’s bioecological theory and previous research in this area: Hypothesis 1 (H1): Parenting styles influence child internet addiction. Specifically, authoritative parenting is negatively associated with internet addiction, while authoritarian and permissive parenting are positively associated with online addiction. Hypothesis 2 (H2): Active mediation in parenting reduces the level of internet addiction. Hypothesis 3 (H3): Active mediation in parenting mediates the effect of parenting style on child internet addiction.

## 4. Methods

### 4.1. Data

Data for this study were collected using a convenience sampling method, which allowed for the recruitment of participants who were readily accessible and willing to participate. In April 2024, survey invitations were distributed to parent groups affiliated with four different schools located in Guangzhou and Macau. These schools represented a diverse range of educational levels, including elementary, middle, and high school, ensuring that the study captured perspectives from parents of children at various developmental stages. A total of 808 parents were invited to participate in the survey. By the end of May 2024, 609 parents had responded. To maintain the integrity of the dataset, seven incomplete surveys were excluded from the analysis, resulting in a final sample size of 600 parents, yielding a response rate of approximately 74.3%.

The demographic characteristics of the participating parents revealed that their average age was 40 years, with a broader age range spanning from 28 to 59 years. The majority of respondents (67%) were female, reflecting the common trend in parent-focused research where mothers are more likely to participate. Additionally, data were collected on the focal children for whom these parents provided responses. The average age of the children was 10.6 years, with ages ranging from 6 to 18 years. In terms of gender distribution, approximately 57% of the children were male. Ethical considerations were carefully addressed in this study. The research protocol underwent a rigorous review process and was approved by the Ethics Review Committee at one of the co-authors’ affiliated universities in Macau. This approval ensured that the study adhered to ethical guidelines for conducting research involving human participants, including obtaining informed consent from all respondents and maintaining the confidentiality of their responses.

### 4.2. Measures

Internet addiction was measured using the parent version of the Chen Internet Addiction Scale (CIAS) [34], which consists of 26 items. The scale assesses the core symptoms of internet overuse, including tolerance, compulsiveness, and withdrawal symptoms, as well as problems related to excessive internet use, such as neglect of family and social life, compromised work or study performance, concealment of use, and physical discomforts (e.g., headaches, sleep disturbances, and backaches). Each item is rated on a scale from 1 (strongly disagree) to 4 (strongly agree). The internet addiction score is calculated as the mean of the item responses, with lower scores indicating a lower level of internet addiction and higher scores reflecting more noticeable addiction. In this study, the scale demonstrated high reliability, with a Cronbach’s alpha of 0.97.

Parenting styles were assessed using the Parenting Styles and Dimensions Questionnaire (PSDQ) [35,36], a 32-item parent-report measure grounded in Baumrind’s framework of authoritative, authoritarian, and permissive parenting styles. Each item is rated on a 5-point Likert scale (1 = Never, 5 = Always), with higher scores reflecting more frequent use of the specified parenting behaviors. In this study, each parenting score is calculated as the mean of the item responses, with higher scores indicating a higher frequency of the parenting style. The PSDQ demonstrated good internal consistency for each of the scales in this study: authoritative (α = 0.91), authoritarian (α = 0.86), and permissive (α = 0.75).

Parental active mediation was measured using the Parenting Mediation in Child Internet Use scale [37], which consists of 8 items of active mediation. Responses were rated on a four-point Likert scale (4 = often, 1= never). The active mediation score is calculated as the mean of the 8 item responses, with higher scores indicating a higher active mediation. The scale demonstrated good reliability in this study, with a Cronbach’s alpha of 0.88.

### 4.3. Statistical Approach

The data analysis process involved multiple stages to ensure a thorough examination of the relationships among key study variables. First, descriptive statistics were computed for all study variables, including means, standard deviations, and frequency distributions, to provide an overview of the data characteristics. Additionally, bivariate correlations were analyzed to assess the fundamental relationships between key constructs, such as parenting style, parental mediation, and internet addiction. These correlations helped identify initial patterns and associations that informed the subsequent analysis. Following the descriptive and correlation analyses, we conducted a structural equation modeling (SEM) analysis to investigate the complex relationships among parenting style, parental mediation, and internet addiction. SEM is a robust statistical technique that allows for the simultaneous estimation of multiple relationships within a theoretical framework. This approach enabled us to test both direct effects—such as the impact of parenting style on internet addiction—and indirect effects, where parental mediation might serve as a mediator in this relationship. By incorporating both direct and indirect pathways, SEM provided a more comprehensive understanding of how these psychological and behavioral factors interact and contribute to the development of internet addiction in children and adolescents. To ensure the reliability and accuracy of our findings, all statistical analyses were conducted using STATA software, version 16.0. The use of SEM in this study provided valuable insights into the mechanisms underlying internet addiction and the role of parenting practices in shaping children’s online behaviors.

## 5. Results

Table 1 presents the descriptive statistics of the study sample. The mean level of internet addiction was 2.23, on a scale from 1 to 4, indicating a moderate degree of internet addiction. With respect to parenting style, the average score for authoritative parenting was 3.66, while authoritarian and permissive parenting had mean scores of 2.40 and 2.82, respectively. These scores were measured on a scale from 1 to 5, suggesting that authoritative parenting was the most commonly reported style among the sample, while authoritarian parenting was relatively the least prevalent. Active mediation in parenting had a mean score of 2.96, ranging from 1 to 4. The average age of the parents in the sample was 40 years, with the majority being mothers (67%). Regarding the children, 57% were boys, and their average age was 10.6 years.

The correlation results for the key variables are presented in Table 2. In line with the previous literature and our proposed hypotheses, internet addiction was negatively correlated with authoritative parenting (r = −0.15, *p* < 0.001) and positively correlated with both authoritarian (r = 0.25, *p* < 0.001) and permissive parenting (r = 0.25, *p* < 0.001). Parental active mediation was also negatively associated with internet addiction (r = −0.15, *p* < 0.001) and positively correlated with authoritative parenting (r = 0.43, *p* < 0.001).

**Figure 1:**
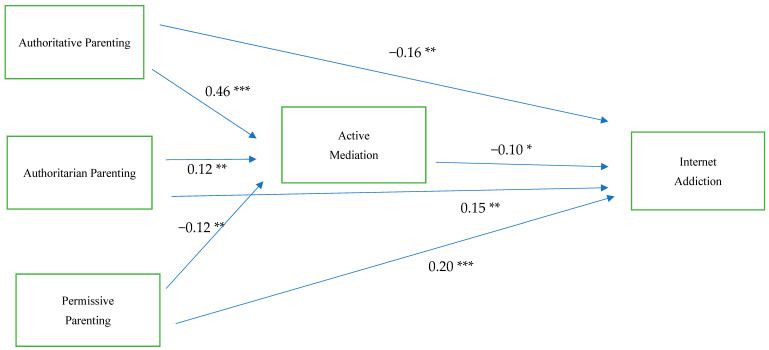
Standardized estimates of parenting style and parental mediation on internet addiction. n = 600, * *p* < 0.05, ** *p* < 0.01, and *** *p* < 0.001.

In Figure 1 and Table 3, we present the standardized coefficients derived from the structural equation modeling (SEM) analysis, providing insights into the relationships among parenting styles, parental mediation, and internet addiction. First, in terms of the relationship between parenting styles and active mediation, the findings indicate a strong positive relationship between authoritative parenting and active mediation (β = 0.46, *p* < 0.001), suggesting that parents who adopt an authoritative parenting style—characterized by warmth, responsiveness, and clear communication—are more likely to engage in active mediation of their children’s internet use. This suggests that authoritative parents tend to be more involved in guiding their children’s online activities, setting appropriate boundaries, and discussing potential risks and benefits. Interestingly, authoritarian parenting also shows a significant positive association with active mediation (β = 0.12, *p* < 0.01), albeit to a lesser extent. This indicates that even parents who employ a more rigid and controlling parenting style may still engage in active mediation, possibly through rule-setting and monitoring strategies rather than open discussion. However, permissive parenting demonstrates a negative association with active mediation (β = −0.12, *p* < 0.01), suggesting that parents who are more lenient and indulgent tend to be less engaged in actively guiding their children’s internet use. This could be due to a more hands-off approach, where children are granted greater autonomy with minimal parental oversight. These findings collectively support Hypothesis 1, confirming that different parenting styles are significantly linked to variations in parental mediation strategies.

With respect to parenting styles and internet addiction, the results indicate that authoritative parenting has a protective effect, as it is negatively associated with internet addiction (β = −0.16, *p* < 0.001). This suggests that children of authoritative parents, who receive consistent guidance and support, are less likely to develop problematic internet use behaviors. In contrast, both authoritarian (β = 0.15, *p* < 0.001) and permissive parenting (β = 0.20, *p* < 0.001) exhibit significant positive associations with internet addiction. This finding highlights the potential risks associated with these parenting styles—authoritarian parenting, with its rigid control, may drive children toward excessive internet use as an escape from strict regulations, while permissive parenting, with its lack of boundaries, may lead to unregulated and excessive screen time.

Parental active mediation was found to be negatively related to internet addiction (β = −0.10, *p* < 0.05), supporting Hypothesis 2. This suggests that when parents actively discuss internet use with their children, set guidelines, and engage in open communication, the likelihood of internet addiction decreases. This result underscores the importance of parental involvement in shaping children’s online behaviors and mitigating potential risks. Further analysis revealed that authoritative parenting had an indirect effect on internet addiction through active mediation (β = −0.05, *p* < 0.05), with a total effect of β = −0.21, *p* < 0.001. This indicates that 23.8% of the total effect of authoritative parenting on internet addiction was mediated by active mediation (−0.05/−0.21). This finding supports Hypothesis 3, reinforcing the idea that one of the mechanisms through which authoritative parenting reduces internet addiction is by promoting active parental mediation. In other words, authoritative parents do not just directly reduce problematic internet use in their children; they also foster active mediation practices, which in turn help regulate children’s online behavior. The R-squared value for the active mediation model was 0.20, meaning that 20% of the variance in active mediation could be explained by the different parenting styles. Meanwhile, the R-squared value for the internet addiction model was 0.13, indicating that parenting styles and active mediation together explained 13% of the variance in internet addiction. While these values suggest that other unmeasured factors also play a role in predicting internet addiction, they still highlight the significant contribution of parenting styles and mediation strategies in influencing children’s online behaviors.

## 6. Discussion

The rapid proliferation of digital technologies and their integral role in daily life have ushered in a new era of challenges, particularly concerning the psychological and behavioral health of children. This study sheds light on the complex interplay between parenting styles and internet addiction in children, with a specific focus on Chinese families. Bronfenbrenner’s ecological systems theory provides a foundational perspective for understanding how family environmental context influences child development and behavior [32].

This study’s findings, with relation to parenting styles and internet addiction, identify authoritative parenting as a protective factor against internet addiction, in line with previous studies [18,31]. Authoritative parents, characterized by a balance of warmth and structure, tend to foster healthier emotional and social development in their children. This style encourages open communication and provides children with the tools to navigate digital environments effectively [19,29]. In contrast, authoritarian and permissive parenting styles, marked by either excessive control or a lack of boundaries, are linked to higher levels of online addiction [21,38]. Authoritarian parents may impose strict rules without fostering understanding or critical engagement, leading to rebellion or secretive behaviors in children [39]. Conversely, permissive parents may fail to set necessary limits, leaving children unprotected from the negative consequences of excessive internet use [14,15].

The implications of these findings suggest that parenting style is not merely a background factor; rather, it is a pivotal element in shaping children’s digital behaviors. It highlights the necessity for parents to adopt an authoritative style, characterized by a combination of high expectations and emotional support, to mitigate the risk of internet addiction [16]. Programs aimed at educating parents about effective parenting strategies could significantly benefit families, equipping them with the skills needed to manage their children’s internet use proactively [8,40].

For the mediating role of parental active intervention, the research underscores the critical mediating role of active intervention in the relationship between parenting styles and internet addiction. Active mediation, which involves engaging children in discussions about their internet activities, emerges as a particularly effective strategy for reducing internet addiction. This finding is significant as it emphasizes that the quality of parent–child interactions can be more influential than mere restrictions on internet use. Specifically, this study reveals that authoritative parents who engage in meaningful conversations about internet use are better positioned to steer their children away from addiction compared to authoritarian parents who focus solely on control [12,36].

These insights have implications for developing parenting programs and intervention strategies. Instead of solely focusing on restrictions or monitoring, programs should prioritize teaching parents how to foster open dialogue with their children about their internet experiences [9,18,28]. Workshops and resources that promote active mediation strategies—such as discussing the content children encounter online, setting mutual agreements on internet use, and educating children about potential online dangers—could empower parents to take a more proactive role in guiding their children’s digital habits [5,11,30].

Given the cultural context of this study, it is essential to recognize the unique challenges that families in China face concerning parenting and internet use. In a rapidly urbanizing society where technological integration is prevalent, traditional parenting norms are increasingly challenged [22]. Parents often experience societal pressure to ensure academic success, which may lead to either overprotection or permissiveness [23]. Understanding these cultural dynamics is crucial in tailoring interventions that resonate with the experiences and challenges faced by Chinese families. Parental mediation strategies may vary in effectiveness based on cultural expectations and norms surrounding child-rearing [20]. Policymakers and educators should consider these cultural nuances when designing initiatives to address internet addiction. Programs that respect and integrate Chinese cultural values while promoting effective parenting practices could lead to better engagement and adherence among parents [13,37].

While this study provides valuable insights into the relationship between parenting styles, parental intervention, and internet addiction, it also highlights several areas for future research. Firstly, longitudinal studies could be beneficial in understanding how these dynamics evolve over time, particularly as children transition from childhood to adolescence—a critical period marked by increased autonomy and exposure to digital technologies [1]. Understanding how parenting strategies may need to adapt during these developmental stages would provide deeper insights into maintaining effective parental engagement [35,41]. Secondly, further exploration into the role of socio-economic factors and parental education levels could enhance our understanding of how different demographic factors influence parenting styles and internet use. The current study predominantly focuses on two regions in China, which may limit the generalizability of the findings. Expanding the sample to include diverse socio-economic backgrounds could provide a more comprehensive view of the issue. Lastly, qualitative research could yield rich insights into the lived experiences of parents and children regarding internet use and addiction. Such research could explore how parents perceive their roles in managing their children’s internet use and the challenges they face in implementing effective strategies [26].

## 7. Conclusions

The findings from this study illuminate the significant role of parenting styles and parental intervention in mitigating internet addiction among children in China. Authoritative parenting emerges as a protective factor, while authoritarian and permissive styles contribute to increased risks of addiction. Importantly, this study underscores the value of parental active mediation in shaping children’s internet behaviors. As society grapples with the implications of increased digital connectivity, it is imperative for parents, educators, and policymakers to work collaboratively in promoting effective parenting practices. By fostering a deeper understanding of the dynamics between parenting styles, mediation strategies, and internet addiction, we can better equip families to navigate the complexities of the digital age, ultimately safeguarding the mental health and well-being of children.

## Figures and Tables

**Table 1:** Descriptive statistics of key variables.

Variables	Mean	S.D.
Internet Addiction [1,2,3,4]	2.23	0.62
Parenting Style [1,2,3,4,5]		
Authoritative	3.66	0.59
Authoritarian	2.40	0.57
Permissive	2.82	0.52
Active Mediation [1,2,3,4]	2.96	0.69

*n* = 600.

**Table 2:** Correlation analysis of key variables.

Variables	1	2	3	4	5
1. Internet Addiction	---				
2. Authoritative Parenting	−0.15 ***	---			
3. Authoritarian Parenting	0.25 ***	0.03	---		
4. Permissive Parenting	0.25 ***	0.22 ***	0.55 ***	---	
5. Active Mediation	−0.15 ***	0.43 ***	0.07	0.04	---

n = 600, *** *p* < 0.001.

**Table 3:** Direct, indirect, and total effects of parenting style and parental mediation on internet addiction.

Predictor	Dependent Variable	Direct Effect	Indirect Effect	Total Effect
Authoritative	Active Mediation	0.46 ***	---	0.46 ***
Authoritarian	Active Mediation	0.12 **	---	0.12 **
Permissive	Active Mediation	−0.12 **	---	−0.12 *
Authoritative	Internet Addiction	−0.16 ***	−0.05 *	−0.21 ***
Authoritarian	Internet Addiction	0.15 **	−0.01	0.14 **
Permissive	Internet Addiction	0.20 ***	0.01	0.21 ***
Active Mediation	Internet Addiction	−0.10 *	---	−0.10 *

n = 600, * *p* < 0.05, ** *p* < 0.01, and *** *p* < 0.001.

## Data Availability

Data available on request due to privacy/ethical restrictions.
